# Physical Activity and Healthy Eating Programming in Schools to Support Student’s Health-Related Fitness: An Observational Study

**DOI:** 10.3390/ijerph182111069

**Published:** 2021-10-21

**Authors:** Timothy J. Walker, Derek W. Craig, Andjelka Pavlovic, Shelby Thiele, Breanna Natale, Jacob Szeszulski, Laura F. DeFina, Harold W. Kohl

**Affiliations:** 1 Center for Health Promotion and Prevention Research, Department of Health Promotion and Behavioral Sciences, The University of Texas Health Science Center at Houston School of Public Health, 7000 Fannin St., Houston, TX 77030, USA; Derek.W.Craig@uth.tmc.edu (D.W.C.); Jacob.Szeszulski@uth.tmc.edu (J.S.); 2 Division of Youth Education, The Cooper Institute, 12330 Preston Road, Dallas, TX 75230, USA; apavlovic@cooperinst.org (A.P.); sthiele@cooperinst.org (S.T.); bnatale@cooperinst.org (B.N.); ldefina@cooperinst.org (L.F.D.); 3 Department of Epidemiology, Human Genetics and Environmental Sciences, The University of Texas Health Science Center at Houston School of Public Health, Austin Regional Campus, Austin, TX 78701, USA; Harold.W.Kohl@uth.tmc.edu; 4 Department of Kinesiology and Health Education, The University of Texas at Austin, 1616 Guadalupe, Austin, TX 78701, USA

**Keywords:** physical activity, nutrition, schools, healthy eating, implementation, physical fitness, children

## Abstract

Centers for Disease Control (CDC) guidelines recommend schools use a coordinated health approach to support healthy eating and physical activity. This study examines whether the number of healthy eating and physical activity programs and activities used by schools and their perceived success relate to students’ health-related fitness. This observational study used data from the Healthy Zone Schools Program. Data (collected in 2017–2019) were integrated from three sources: (1) school surveys, (2) FitnessGram^®^, and (3) the Texas Education Agency. Independent variables were the number of health promotion programs and activities and their perceived success; dependent variables were meeting Healthy Fitness Zone Standards (HFZ) for aerobic capacity and body mass index (BMI). We used mixed-effects logistic regression models. Fifty-six schools were in the analytic sample (*n* = 15,096 students with aerobic capacity data and *n* = 19,969 with BMI data). Results indicated the perceived success of physical activity programs/activities was significantly associated with students meeting HFZ standards for aerobic capacity (OR = 1.32, CI = 1.06–1.63). There was a significant direct association between the number of physical activity and healthy eating activities implemented (OR = 1.04, CI = 1.01–1.06) and students meeting HFZ for BMI. Schools using multiple health programs and activities need to balance the number provided with their capacity to maintain success.

## 1. Introduction

Improving children’s physical activity and nutrition are two public health priorities in the United States [1]. Only about 25% of children (6–17 years old) participate in the recommended 60 min per day of moderate-to-vigorous aerobic physical activity [2]. Additionally, many children consume an unbalanced diet that does not align with key dietary guidelines; 60% of U.S. children consume fewer fruits, and 93% consume fewer vegetables than recommended [3]. Inadequate physical activity and a poor diet can negatively impact children’s weight status, metabolic, and cardiovascular health. Given the short- and long-term impacts of children’s health behaviors, there is a pressing need to help children adopt and maintain healthy lifestyles.

Children’s health behaviors are influenced by various societal sectors, including families, schools, and communities [4]. Schools are uniquely positioned to support children’s health for multiple reasons. First, children spend the majority of their waking hours in school [5]. Additionally, schools have an existing infrastructure (e.g., physical education, recess, food services, health classes, and on-site before- and after-school programming) that can directly support students’ health behaviors. Lastly, school-based programming can reach a wide range of youth throughout their primary and secondary education and students from diverse racial/ethnic, socioeconomic, and geographic backgrounds.

Given the important role schools play in health promotion, the Centers for Disease Control and Prevention (CDC) has put forth school health guidelines to promote healthy eating and physical activity [6]. The guidelines reflect a coordinated school health approach and include strategies to aid implementation. For example, the guidelines recommend schools establish supportive environments for healthy eating and physical activity by providing access to healthy foods and physical activity opportunities and promoting healthy behavior choices through marketing techniques and the use of student rewards [6].

Another school health guideline recommendation is to implement a comprehensive physical activity program. The use of comprehensive programs aligns with other coordinated approaches such as a Comprehensive School Physical Activity Program (CSPAP) and a whole-of-school approach [7,8]. Both a CSPAP and whole-of-school approach set out to establish school environments that provide ≥60 min per day of moderate-to-vigorous physical activity. To achieve this goal, schools must coordinate resources and efforts to offer regular physical education, physical activity opportunities during the school day (e.g., recess and classroom-based activity), and before- and after-school programming. In addition, these approaches recognize the importance of staff involvement and engagement from the broader community (e.g., families and community partnerships). Overall, to achieve school health guideline recommendations, schools need to implement multiple programs throughout the year, which requires a comprehensive and coordinated approach.

Using a comprehensive and coordinated health promotion approach in schools presents numerous implementation challenges. Schools need support from multiple stakeholders (e.g., school leaders, teachers, staff, and parents), knowledge of existing programs, motivation, and the capacity to implement them [9,10]. At the school level, common implementation barriers to health promotion efforts include a lack of resources (both staffing and financial), a crowded school curriculum, and poor implementation climate [11,12,13,14]. These barriers can limit the adoption of evidence-based approaches and influence implementation success. As a result, decision makers often need to weigh the benefits of offering multiple health programs and their ability to implement them with quality and success in a limited resource environment.

The Healthy Zone School Program (HZSP) (https://www.healthyzoneschool.com/ (accessed on 27 August 2021)) was developed to help schools in the Dallas, Texas, area coordinate their health promotion efforts and to establish environments that support physical activity and healthy eating behaviors [15]. The HZSP assists schools by providing access to health promotion resources, guidance about existing programs and activities, school-to-school mentorship, and implementation guidance. By participating, schools are able to offer a variety of healthy eating and physical activity programs for their students. However, to help schools improve programmatic decision making, more information is necessary to understand how the use of multiple programs relates to key student health-related outcomes such as cardiorespiratory fitness and body mass index (BMI).

The network of schools in the HZSP provides a unique opportunity to examine how the number and success of healthy eating and physical activity programs relate to students’ health-related fitness. More specifically our aims are two-fold. First, we aim to examine whether the number of healthy eating and physical activity programs used and their perceived success relate to students’ cardiorespiratory fitness. Second, we aim to examine whether the number of programs and their perceived success relate to students’ BMI.

## 2. Methods

### 2.1. Study Design and Setting

The HZSP enrolls a cohort of schools annually and works with them for three years. Data for this observational study were from schools in the HZSP between the years 2017 and 2019 (Cohorts 6–8). We integrated data from three sources: (1) school surveys, (2) FitnessGram^®^, and (3) publicly available data from the Texas Education Agency (TEA). As part of HZSP participation, a school representative completed a survey in the spring of each year (April–May). These surveys were retrospective in nature, meaning they included questions about health promotion programs and activities offered throughout the respective school year. Schools also provided access to FitnessGram^®^ data, which is a set of validated field tests used to assess students’ health-related fitness [16]. We also integrated school characteristics data obtained from the TEA website [17]. All data for this study were collected from September to May during each school’s first year with the HZSP. The Committee for the Protection of Human Subjects at the University of Texas Health Science Center at Houston (HSC-SPH-18-0549) and the Institutional Ethics Review Board at the Cooper Institute approved this study.

### 2.2. HZSP

The HZSP was developed to help schools create a sustainable health environment by adopting and implementing programs and activities to promote physical activity and healthy eating [15]. The program provides the following: general guidance to schools, access to specific programs and activities, access to funding, training for teachers, and school-to-school mentors (Table 1). For purposes of this study, programs were defined as initiatives that consist of a planned series of events such as a school running club or school garden program. Activities were defined as one-time events that do not require continued participation over time such as a field day or cafeteria taste testing of healthy foods. A comprehensive list of the programs and activities that were promoted as part of the HZSP is provided in Table 2.

### 2.3. Participants

Schools in the Dallas, Texas (USA), metropolitan area were eligible to apply for the HZSP. Program staff recruited schools by making presentations at district health advisory committee meetings and advertising on social media. The HZSP was available to elementary, middle, and high schools, including public, private, and charter schools. Program staff from the Cooper Institute and United Way of Metropolitan Dallas accepted applications and selected schools for participation based on their health-related needs and policies. Schools were enrolled on an annual basis (a new cohort for each year) and agreed to participate in the HZSP for three years. As part of their participation, schools designated a staff member (usually a physical education teacher) to complete an annual survey (details explained below) and to collect annual FitnessGram^®^ data. During the time of the study, there were 61 schools that were actively participating in the HZSP, serving about 44,000 students in total. For inclusion in this study, schools needed to have completed the HZS survey and entered their students’ fitness data into the FitnessGram^®^ software in the spring of their first year with the program.

### 2.4. Measures

HZSP Survey: We used HZSP questionnaire data for our independent variables, which were the number of programs, number of activities, and their perceived success. The HZSP survey included a series of yes/no questions about each school’s use of physical activity and healthy eating programs and activities (Table 2). We created six variables based on a series of questions: (1) number of total programs (physical activity and healthy eating); (2) number of total activities (physical activity and healthy eating); (3) number of physical activity programs; (4) number of physical activity activities; (5) number of healthy eating programs; (6) number of healthy eating activities.

The survey also included questions about the respondent’s perceived success of each respective program and activity. More specifically, if a respondent answered “yes” to using a program (or activity), they received a follow-up question asking them to indicate the program’s “overall success” using a 1–10 scale (1 = not at all successful and 10 = extremely successful). We created a series of perceived success variables that corresponded with the previously mentioned program and activity variables. Because the perceived success scores for programs and activities were highly correlated (r = 0.68), we created a weighted average to represent perceived success for: (1) all programs/activities, (2) physical activity programs/activities, and (3) healthy eating programs/activities. Lastly, the survey included a question asking whether the school provided scheduled recess during the school year (yes/no), which we also considered as a control variable.

FitnessGram^®^ Data: FitnessGram^®^ data for all participating schools were collected annually in the second half of the school year during their time in the HZS program. The two dependent variables were students’ meeting Healthy Fitness Zone (HFZ) standards for cardiorespiratory fitness and body mass index (BMI). Cardiorespiratory fitness was assessed for each student using one of three methods: 20-m pacer test, 15-m pacer test, or a one-mile run test. The test results (number of pacer laps and one-mile run times) were entered in the FitnessGram^®^ software by a Physical Education teacher or designated staff. The software calculated predicted aerobic capacity values using regression equations [16,18]. Predicted aerobic capacity values were reported as estimates of maximal oxygen uptake (V0_2max_), and these values were used to determine whether a student was meeting the criterion-referenced HFZ standard, which represents a fitness level associated with health benefits. The HFZ standards are organized by age- and sex-specific cut points, allowing for meaningful comparisons across the sample of students [16,19]. The HFZ standards apply to students ≥9 years of age in the 3rd grade or above. Thus, data were excluded if: (1) students were too young; (2) the aerobic capacity test was invalid (e.g., >190 laps reported); (3) the test was not performed in the winter/spring of the school’s first year with the program. BMI was also assessed for each student. Physical Education teachers entered height and weight information into the FitnessGram^®^ software, where it was converted by the software into BMI values using standard equations. HFZ standards for BMI were determined by using the CDC age- and sex-specific values associated with health benefits [20].

TEA Data: We used TEA data to obtain school characteristics to control for in the subsequent analysis. We included the following variables: school type (elementary, middle, high school), enrollment (total number of students), Title 1 status (serving ≥40% of students who were economically disadvantaged), percentage of English language learners, and percentage of students in respective race/ethnicity categories (Black, Hispanic, White, or another race). Because most schools in the program were elementary schools, we collapsed the middle and high school categories for school type in the analysis. We also created a variable representing each school’s student racial/ethnic composition by categorizing them into one of four exclusive groups: (1) majority White (≥50%); (2) majority Black (≥50%); (3) majority Hispanic (≥50%); and (4) diverse (no single race/ethnicity group ≥50%). These variables were selected because they represent school characteristics that may influence the adoption and implementation of programs and activities and have evidence of being associated with health-related fitness outcomes [21].

### 2.5. Statistical Analysis

We first examined descriptive statistics (e.g., means, frequencies, distributions) for both the individual-level (e.g., sex, age, and grade) and school-level variables (e.g., school type, cohort, enrollment). We then examined Pearson correlations between the continuous school-level variables and frequency tables for categorical variables. We also calculated the intraclass correlation coefficient (ICC) for each dependent variable (aerobic capacity and BMI) to determine the variance explained by differences between schools.

We used the physical-activity-specific program and activity variables when examining associations with meeting HFZ standards for aerobic capacity (Aim 1) and all program/activity type variables (both physical activity and healthy eating) when examining associations with BMI (Aim 2). We used a series of mixed-effects logistic regression models for both study aims. First, we examined associations between each school characteristic variable (e.g., school type, number of students) and each dependent variable (meeting HFZ for aerobic capacity and BMI) while controlling for individual-level variables (e.g., sex and age). These models were used to determine which school characteristic variables would be controlled for in the final models. School characteristics that were significantly related to meeting HFZ standards were included in final models for each respective outcome. We included sex as a random coefficient in all models. We used Stata 15.1 (StataCorp, College Station, TX, USA) for the analysis and *p* < 0.05 to represent statistical significance.

## 3. Results

Fifty-six (of 61) schools completed FitnessGram^®^ data for the winter/spring of their first year with the HZS program. From the participating schools, there were a total of 21,240 students across grades 3–12. For aerobic capacity, 6144 students were excluded from the analysis because they were: (1) too young for the test (*n* = 3770), (2) had an incomplete test (*n* = 1589), (3) were excused by the teacher from the test (*n* = 231), or (4) had an invalid score (*n* = 554). Thus, there were 15,096 students in the analytic sample for aerobic capacity. For BMI, 1271 students were excluded from the analysis because they had incomplete/missing data (*n* = 602) or were excused by the teacher from the test (*n* = 669). Thus, there were 19,969 students in the analytic sample for BMI. For both samples, about 52% of students were boys, the average age was 11 or 12 years old, and 50–59.5% were from elementary schools (Table 3).

All schools included in the analysis were public schools (*N* = 52) or public charter schools (*N* = 4), 78.6% were elementary-level, and 55.4% were Title 1 (Table 4). Cohort 6 had the most schools, which included schools that started the program in the fall of 2017. Schools served an average of 718 students, and almost half the schools were serving a diverse community, meaning no single race/ethnicity was greater than 50% (Table 4). On average, schools offered a total of 9.5 physical activity and healthy eating programs and 8.0 activities throughout their first year with the HZS program. Schools tended to offer more physical activity than healthy eating activities and a similar number of programs for each respective target behavior (Table 4).

### 3.1. Aerobic Capacity Results

The ICC for meeting HFZ standards for aerobic capacity was 0.26, indicating about 26% of variance in students meeting HFZ standards was explained by differences in schools. When examining school-level variables separately (while controlling for individual-level variables), enrollment, Title 1 status, English language learners, and school-level race/ethnicity were all related to students meeting HFZ standards for aerobic capacity. The specific odds ratios were: enrollment (per 100 students) OR = 0.94, CI = 0.90–0.98; Title 1 status OR = 0.45, CI = 0.28–074; the percentage of English language learners OR = 0.98, CI = 0.98–0.99; and school-level race/ethnicity OR = 0.35, CI = 0.12–0.99 for majority Black and OR = 0.33, CI = 0.18–0.60 for majority Hispanic (referent group was diverse schools).

Model results revealed the total number of physical activity programs and activities provided by schools were not significantly associated with meeting HFZ standards for aerobic capacity (OR = 0.99, CI = 0.83–1.13; OR = 1.06, CI = 0.93–1.22, respectively). However, the average perceived success scores across the total number of physical activity programs and activities were significantly associated when holding other variables constant (OR = 1.32, CI = 1.06–1.63). Results indicated a 1-unit increase in average perceived success was associated with 32% higher odds for students meeting HFZ standards for aerobic capacity.

### 3.2. BMI Results

The ICC for meeting HFZ standards for BMI was 0.04, indicating about 4% of the variance in students meeting HFZ standards was explained by differences in schools. When examining the school-level variables separately, Title 1 status (OR = 0.57, CI = 0.48–0.67), the percentage of English language learners (OR = 0.99, CI = 0.98–0.99), and school-level race/ethnicity (OR = 0.63, CI = 0.45–0.88 for majority Black; OR = 0.53, CI = 0.45–0.62 for majority Hispanic; referent group was diverse schools) were all related to students meeting HFZ standards for BMI when controlling for individual-level variables (sex and age).

Model results revealed the number of programs was not related to students meeting HFZ for BMI. However, there was a significant direct association between the number of activities implemented (OR = 1.04, CI = 1.01–1.06) and students meeting HFZ for BMI. These results indicate that for each additional activity implemented, the odds were 4% higher for students meeting HFZ standards for BMI. The perceived implementation success of programs/activities was not significantly related to students meeting HFZ standards for BMI (Table 5). When examining physical activity and healthy eating programs and activities separately, there was a significant association between the number of physical activity activities and students meeting HFZ standards for BMI (OR = 1.05, CI = 1.02–1.08) but no significant associations for healthy eating programs or activities (Table 5).

## 4. Discussion

This study examined whether physical activity and healthy eating programs and activities offered by the HZSP were associated with students’ health-related fitness. Aim 1 study results suggest physical activity program/activity success was associated with students meeting HFZ standards for aerobic capacity. However, there was no evidence of an association between the total number of physical activity programs or activities offered and students’ aerobic capacity. These findings suggest school-based efforts to support students’ cardiorespiratory fitness may benefit by focusing on the success of programs and activities in addition to providing access.

Program success can be impacted by a school’s ability to select effective programs and implement them with fidelity [22]. Implementing multiple programs can be difficult because of the need to coordinate stakeholders, identify effective programs, and use effective implementation strategies [9]. By participating in the HZSP, schools received strategies (e.g., ways to promote programs, approaches to incentivize participation) for fostering a culture of health from the Cooper Institute and their respective School Wellness Committee. Schools with greater levels of program/activity success may have delivered programs more effectively to improve children’s physical activity levels and, in turn, cardiorespiratory fitness. These findings highlight the potential value of choosing effective programs that can be implemented with quality.

In order to support student health, schools need to provide opportunities for physical activity and healthy eating. However, the number of physical activity programs/activities provided by schools was likely not related to students meeting HFZ standards for cardiorespiratory fitness for multiple reasons. First, on average, schools offered about 4.7 physical activity programs (range: 1–8) and 5.8 activities (range: 1–9). Within the study sample, the lowest combined number of physical activity programs and activities offered by a school was three. Thus, it is possible that when offering ≥3 physical activity programs and activities, extra programming may not provide additional benefits to students’ cardiorespiratory fitness.

Another reason is the variation in program/activity type and impact. For example, programs developing self-management strategies to support health (e.g., Go Slow Whoa) may benefit children through intrinsic behavior change, whereas providing access (e.g., open gym time) may facilitate activity for those already motivated to be active. In both cases, the programmatic impact is unclear. Impact is defined as reach (the number of students who participated in a program/activity) × efficacy (how much a program/activity improved students’ cardiorespiratory fitness) [23]. Thus, implementing a high number of low-impact programs/activities may not contribute to students’ fitness levels more than implementing a low number of high-impact programs/activities.

In contrast to Aim 1 findings, our results for Aim 2 indicated there was a modest (yet significant) association between the total number of health promotion activities and students meeting HFZ standards for BMI. More specifically, this association appeared to be driven by the number of physical activity activities that schools provided. School-based approaches targeting students’ BMI levels have had mixed success [24]. This is in part due to variations in program type (e.g., multicomponent versus single component programs), program duration, the target population, and measures used to examine outcomes. Overall, there are no clear trends indicating which program characteristics are associated with program effectiveness. Our findings suggest that having multiple activities to promote physical activity and healthy eating behaviors throughout the school may support healthier BMI levels among students.

Given activities are one-time events, it is unlikely that a single activity would impact students’ BMI levels. However, providing multiple activities may be beneficial because they can help contribute to a school environment that supports healthy eating and physical activity. The CDC’s school health guidelines recommend schools establish a climate that encourages healthy eating [6]. Thus, providing multiple healthy eating activities throughout the year may support a school’s health climate, which has the potential to positively impact students’ health behaviors. Neither the number of programs nor their perceived success were related to students meeting HFZ standards for BMI. There are multiple reasons for this finding, which include: (1) the number of programs variable does not account for varying levels of program impact, and (2) BMI levels tend to be challenging to influence through school-based programs alone.

### 4.1. Limitations

This study was conducted as part of a larger evaluation effort, which has implications to consider when interpreting study results. First, this was an observational study, which limits causal conclusions as it is possible that the perceived success of programs/activities is due to students having better fitness. Second, the sample included schools that were selected into the HZSP program based on their desire to improve health outcomes. This enrollment approach introduces a potential selection bias, given that the sample of schools may have more supportive health promotion environments than the general population of schools. Further, the sample consisted mostly of public elementary schools, which limited our ability to compare across school types and limits the generalizability of findings. Third, the program and activity data were from a self-reported survey completed by a single school representative. Thus, it is possible survey respondents over- or underreported programs/activities offered or their corresponding level of success. Additionally, the survey did not include questions about the school’s compliance with physical activity policies or physical education, which are confounding factors. Last, the program/activity success variable was aggregated across the success scores for each program and activity. Thus, it represents a broader measure of program success, which lacks validity and reliability testing.

### 4.2. Strengths

This study provides important information about enhancing school environments through health promotion programs and activities. One study strength is the integration of student-level fitness data, school-level information about health promotion programs and activities, and school characteristics obtained from TEA. These unique data sources allowed us to examine how school-level programs related to students’ health-related fitness. This study is also unique in that it examined the use of programs and activities in a real-world environment. The HZSP provided access to programs/activities and resources to support their use and implementation. As part of the HZSP, schools were responsible for choosing which programs and activities to use and overseeing implementation. As a result, there was no research staff aiding with program delivery efforts and thus introducing a potential delivery agent bias [25]. Therefore, the information from this study can be useful to schools outside of the HZSP that are balancing decisions about providing the right number of programs and activities and their ability to implement them with success.

This study also takes a broader perspective by examining multiple programs and activities implemented throughout the school year. Many programs initially undergo efficacy and effectiveness testing, where the goal is to assess the program’s impact under ideal conditions [26]. However, given the nature of the HZSP, we were able to examine how the use of multiple programs and activities relates to student health in a real-world setting. Additional research is necessary to further understand whether certain combinations of programs and activities may be most effective. A better understanding about specific programming can help schools use resources in effective and efficient ways to have the greatest impact on student health.

## 5. Conclusions

Schools play an important role in providing access to programs and activities to support student health. Study findings indicate an association between physical activity program/activity success and students’ cardiorespiratory fitness. This has two important implications. First, program planners should consider both program/activity quality and implementation feasibility when selecting programs and activities. Second, school leaders and staff may benefit by improving their school’s capacity to adopt, implement, and maintain programs effectively [27,28]. Improving implementation capacity can help improve the use of school resources (including staff time and funding) and establish school environments that can better sustain health promotion programming.

Study findings also suggest the number of health promotion activities provided are associated with students meeting HFZ standards for BMI. Thus, program planners should consider offering multiple activities throughout the school year while also balancing program/activity success. More research is necessary to better understand how multiple programs and activities can be used together to best support student health. With this information, school staff may consider choosing programs based on impact, implementation feasibility, and how they fit with their schools’ other programming efforts.

## Figures and Tables

**Table 1:** Programmatic elements of the Healthy Zone Schools Program.

Healthy Zone School Program Element	Description
Training for teachers	Each fall, schools attend orientation training to learn about the successful implementation of HZS.
Guidance to schools	Annually, school wellness committees meet with the program staff to determine goals and select programs/activities.
Access to activities	A list of one-time events to promote health (e.g., family fitness night, community walk, cafeteria taste testing).
Access to programs	A list of high-quality ongoing programs to promote health (e.g., running club, in-class activity breaks, school garden).
Access to funding opportunities	Schools receive annual funding to support activities and programs.
Peer-to-peer mentors	The orientation training helps facilitate networking and mentorship between new and returning schools.

**Table 2:** Programs and activities that were part of the Healthy Zone Schools Program.

Program Name	Program Description	Primary Target Outcome(s)
Kids’ Heart Challenge	Students learn about heart health while raising money for the American Heart Association.	Physical Activity
In-Class Activity Breaks	Physical activity breaks provided during classroom instruction time (e.g., GoNoodle).	Physical Activity
Running club	Youth running groups before or after school (e.g., Marathon Kids, school run club)	Physical Activity
Open gym time	Community access to school gym for games and exercise.	Physical Activity
Non-School Participation/School Camps	Access to programs such as: cheerleading, drill team, musical/theater activities, future farmers of America, etc.	Physical Activity
Let’s Move Active Schools	An initiative targeting childhood obesity by creating healthy environments, encouraging proper nutrition, and providing opportunities for physical activity.	Physical Activity and Healthy Eating
Alliance for Healthier Generation	An initiative promoting physical and mental wellbeing of children and adolescents.	Physical Activity and Healthy Eating
Healthier US School Challenge	Provides recognition to schools that promote healthy eating and physical activity.	Physical Activity and Healthy Eating
NFL Play 60 Challenge	Encourages students to log daily activity over a 4-week period.	Physical Activity
Go Slow Whoa	This program teaches youth how to recognize healthy options (GO), what they should have in moderation (SLOW), and what they should avoid or consume minimally (WHOA).	Healthy Eating
School Garden	On-site garden for students to plant and harvest produce to learn about gardening and healthy eating.	Physical Activity and Healthy Eating
Healthy celebrations	For school-related celebrations, foods of minimal nutritional value are not served.	Healthy Eating
Water drinking promotion	Students and staff are encouraged to reduce the number of sugary drinks they consume while increasing their water consumption.	Healthy Eating
Food logging	The school community tracks the types of foods they are consuming for a designated time period (e.g., 21-day Challenge).	Healthy Eating
Fuel up to Play 60	An in-school nutrition and physical activity program that encourages youth to consume nutrient-rich foods and achieve ≥60 min of physical activity every day.	Physical Activity and Healthy Eating
Online Nutritional Program	Online programs that teach youth about healthy eating patterns.	Healthy Eating
General Health Signage	Signage throughout school to promote and educate about fitness, physical activity, nutrition and healthy living.	Physical Activity and Healthy Eating
Health or wellness class	A class offered during the school day outside of Physical Education where students learn skills to live a healthy lifestyle.	General Health
Grab and Go Breakfast	Access to quick and nutritious meals for students. The items are displayed by the cafeteria or at school entrances so that they are easily accessible.	Healthy Eating
**Activity Name**	**Activity Description**	**Target Outcome**
Field day	A full-day event for students to participate in sporting events and other physical activities.	Physical Activity
Family Fitness Night	An after-school event for families to participate in fun games and activities with their child.	Physical Activity
Pedometer Challenges	Teacher sets up challenges for students to complete a certain number of steps.	Physical Activity
Walk/Ride to school	Designated day for students to walk or ride their bikes to school with the purpose of increasing physical activity.	Physical Activity
Community or school run/walks	Schools select a local race for students, staff, and families to participate in by running or walking (e.g., fun run, 5K races, etc.).	Physical Activity
Walk-a-thon or Community walking event	Planned walking event for students, families and schools to raise money or awareness for a cause (e.g., walk for diabetes, walk for heart health, cause-centered 5K races, etc.)	Physical Activity
Healthy food cooking competition	A cooking competition for students and staff to make healthy recipes that are simple and cost effective.	Healthy Eating
Veggie fruit promotion	Schools provide opportunities for students to try new fruits and vegetables through promotional activities such as Fresh Fruit Friday, Harvest of the Month, etc.	Healthy Eating
Cafeteria taste testing of healthy foods	School nutrition offers a taste test allowing students to experience and vote on new healthy food items that could be added to school menus.	Healthy Eating
Health fair	An event for local businesses (e.g., grocery store, dentist, gym) where they can provide health-related information, screenings, and activities to families.	General Health
General Health Awards *	Student and/or teacher recognition for exceptional contribution towards creating a healthy school environment.	General Health
Family activity challenges	Family challenges that promote fitness and physical activity.	Physical Activity
Newsletters *	Compiled health information and initiatives shared with students, families, and schools.	General Health

*, respondents further specified whether awards and newsletters were for physical activity, healthy eating, and/or general health.

**Table 3:** Descriptive Information for Students by Outcome Variable.

Variable	Aerobic Capacity Sample (*n* = 15,096)	BMI Sample (*n* = 19,969)
Sex (%, *n*)		
Boy	52.3 (7888)	51.8 (10,340)
Girl	47.7 (7208)	48.2 (9629)
Age in years (mean, sd)	12.0 (2.4)	11.4 (2.5)
Grade level (%, *n*)		
Elementary (3–5)	50.1 (7560)	59.5 (11,883)
Middle (6–8)	28.7 (4328)	22.8 (4554)
High (9–12)	21.2 (3208)	17.7 (3532)
Aerobic capacity test type (%, *n*)		
20-m pacer	75.3 (11,365)	n/a
15-m pacer	4.0 (611)	n/a
One-mile run	20.7 (3120)	n/a

BMI, body mass index; n/a, not applicable.

**Table 4:** Descriptive Information for Healthy Zone Schools (total sample = 56 schools).

School Characteristics	
School level (%, *N*)	
elementary	78.6 (44)
Non-elementary	21.4 (12)
Cohort (%, *N*)	
6 (began program fall 2017)	42.9 (24)
7 (began program fall 2018)	33.9 (19)
8 (began program fall 2019)	23.2 (13)
Average total number of students (mean, SD)	717.8 (560.7)
Title 1 (%, *N*)	55.4 (31)
Percent English language learnings (mean, SD)	22.6 (23.5)
School race/ethnicity (%, *N*)	
Majority White (≥50%)	17.9 (10)
Majority Black (≥50%)	7.1 (4)
Majority Hispanic (≥50%)	28.6 (16)
Diverse (no single race/ethnicity group ≥50%)	46.4 (26)
**Physical Activity Program and Activity Variables (Mean, SD)**	
Number of physical activity programs (possible range: 0–10)	4.7 (1.7)
Number of physical activity activities (possible range: 0–9)	5.8 (1.9)
Average physical activity program/activity success rating (0–10)	8.3 (1.1)
**Healthy Eating Program and Activity Variables (Mean, SD)**	
Number of HZS healthy eating programs (possible range: 0–12)	5.0 (1.6)
Number of healthy eating activities (possible range: 0–5)	2.5 (1.6)
Average healthy eating program/activity success rating (0–10)	8.0 (1.3)
**Total Program and Activity Variables (Mean, SD)**	
Number of total programs (possible range: 0–19)	9.5 (2.4)
Number of total activities (possible range: 0–13)	8.0 (2.4)
Average activity success rating (0–10)	8.2 (1.2)

**Table 5:** Main effects model results (OR (95% CI)).

Variable	Meeting HFZ Standards for BMI (*n* = 19,969)	Meeting HFZ Standards for Aerobic Capacity (*n* = 15,096)
All Program/Activity Models		
All programs	1.00 (0.98–1.02)	N/A
All activities	1.04 (1.01–1.06) *	N/A
All program/activity success	1.02 (0.98–1.07)	N/A
Physical Activity Program/Activity Models		
Physical activity programs	0.99 (0.96–1.02)	0.99 (0.83–1.13)
Physical activity activities	1.05 (1.02–1.08) *	1.06 (0.93–1.22)
Physical activity program/activity success	1.02 (0.98–1.07)	1.32 (1.06–1.63) *
Healthy Eating Program/Activity Models		
Healthy eating programs	1.01 (0.98–1.05)	N/A
Healthy eating activities	1.02 (0.96–1.07)	N/A
Healthy eating program/activity success	1.00 (0.97–1.05)	N/A

*, *p* < 0.05;. Aerobic capacity model was adjusted for individual-level variables (sex, age, aerobic test, BMI) and school-level variables (total number of students and Title 1 status). BMI models were adjusted for individual-level variables (sex and age) and school-level variables (Title 1 status, school race/ethnicity composition).

## Data Availability

The FitnessGram^®^ and survey data presented in this study are available on request from the corresponding author and with permission from The Cooper Institute. Data are not publicly available due to privacy and licensing. The School Characteristics data are available from the Texas Education Agency, https://tea.texas.gov/ (accessed on 8 December 2020).
